# Quantitatively assessing aging effects in rapid motor behaviours: a cross-sectional study

**DOI:** 10.1186/s12984-022-01035-1

**Published:** 2022-07-26

**Authors:** Richard Hugh Moulton, Karen Rudie, Sean P. Dukelow, Stephen H. Scott

**Affiliations:** 1 Department of Electrical and Computer Engineering, Queen’s University, Kingston, ON Canada; 2 School of Computing, Queen’s University, Kingston, ON Canada; 3 Ingenuity Labs Research Institute, Queen’s University, Kingston, ON Canada; 4 Department of Clinical Neurosciences, University of Calgary, Calgary, AB Canada; 5 Hotchkiss Brain Institute, University of Calgary, Calgary, AB Canada; 6 Department of Biomedical and Molecular Sciences, Queen’s University, Kingston, ON Canada; 7 Centre for Neuroscience Studies, Queen’s University, Kingston, ON Canada; 8 Department of Medicine, Queen’s University, Kingston, ON Canada

**Keywords:** Rapid motor behaviour, Sensorimotor system, Aging, Robotic assessment

## Abstract

**Background:**

An individual’s rapid motor skills allow them to perform many daily activities and are a hallmark of physical health. Although age and sex are both known to affect motor performance, standardized methods for assessing their impact on upper limb function are limited.

**Methods:**

Here we perform a cross-sectional study of 643 healthy human participants in two interactive motor tasks developed to quantify sensorimotor abilities, Object-Hit (OH) and Object-Hit-and-Avoid (OHA). The tasks required participants to hit virtual objects with and without the presence of distractor objects. Velocities and positions of hands and objects were recorded by a robotic exoskeleton, allowing a variety of parameters to be calculated for each trial. We verified that these tasks are viable for measuring performance in healthy humans and we examined whether any of our recorded parameters were related to age or sex.

**Results:**

Our analysis shows that both OH and OHA can assess rapid motor behaviours in healthy human participants. It also shows that while some parameters in these tasks decline with age, those most associated with the motor system do not. Three parameters show significant sex-related effects in OH, but these effects disappear in OHA.

**Conclusions:**

This study suggests that the underlying effect of aging on rapid motor behaviours is not on the capabilities of the motor system, but on the brain’s capacity for processing inputs into motor actions. Additionally, this study provides a baseline description of healthy human performance in OH and OHA when using these tasks to investigate age-related declines in sensorimotor ability.

**Supplementary Information:**

The online version contains supplementary material available at 10.1186/s12984-022-01035-1.

## Background

The ability to perform rapid motor behaviours underpins our interactions with the world, e.g., driving a car, dancing with a partner, or simply reacting when bumped walking in a crowded shopping mall. In recognition of their importance to our daily lives, motor skills have been incorporated into a number of neuropsychological tests, assessing individuals for cognitive and sensorimotor impairments [1–3]. Unfortunately, motor abilities predictably decline with age and these declines eventually limit many individuals’ independence [4]. With the world projected to have two billion people aged 60 or over by 2050, [5], there is a powerful motivation to measure the effects of aging on the motor system.

Aging impacts individuals’ motor abilities in a number of ways, including: reducing muscle strength [6–8], reducing visuomotor adaptation [9, 10], worsening reach-to-grasp movements [11], declining motor imagery abilities [12], decreasing accuracy in bimanual movements [13], increasing perception of physical fatigue [14], and decreasing proprioceptive acuity [15–17]. There is evidence that an individual’s aging experience will be affected by their sex, with aging having different impacts on various regions in male and female brains [18–20]. There are also underlying sex-related differences in both sensorimotor skill [15, 21] and visuospatial abilities [22–24].

Confoundingly, there is also evidence that some characteristics of the sensorimotor system are resilient to age, such as the mechanical properties of the elbow [25], the ability to perform complex motor actions [26, 27], the ability to act without visual feedback [28, 29], and grip strength when fatigued [30]. This points to the difficulty in deciding how declines in motor abilities due to age will affect daily activities at the population-level, let alone for a given individual.

Therefore, there is a clear need for tests that provide a holistic view of age-related declines in motor abilities. There has been a recent proliferation of rapid motor behavioural tasks with interactive components. To date these tasks have been used to quantify impairments after stroke [31, 32], to study decision-making [33, 34], and to study planning [35], but they also hold the promise of assisting research into the effects of age and sex on sensorimotor skills. Understanding how motor behaviours change with age in these interactive settings will help to develop new neuropsychological tests and equipment to evaluate an individual’s ability to perform every day rapid motor actions.

The purpose of this study is to better understand the effects that age and sex have on an individual’s rapid motor skills. Our hypothesis is that both age and sex will affect rapid motor skills, with participants who are younger and male showing superior performance. We test this hypothesis with two interactive motor behaviour tasks, namely Object-Hit (OH) [31] and Object-Hit-and-Avoid (OHA) [32], which are performed in a robotic exoskeleton to enable the recording of upper limb and joint positions throughout trials. We verify that these tasks are appropriate for testing healthy individuals by demonstrating that participants must reach and maintain their peak steady-state rate of performance to maximize their performance during trials. We then use a large dataset of healthy control participants (n = 643, ages 18–93) who have performed these tasks to assess aging effects on motor behaviours. We perform linear regressions with 16 recorded and computed parameters to determine which are significantly impacted by age. We also tested for sex-related effects (male vs. female) on motor behaviours and on aging effects given their occurrence in the sensorimotor and aging literatures.

## Methods

### Participants

A total of 643 healthy human participants (368 female and 275 male; 64 left-handed, 577 right-handed, and 2 mixed; median age 45) performed the Object-Hit task (Table 1) or the Object-Hit-and-Avoid task (Table 2), with the majority of participants performing both tasks.

**Table 1:** Participant demographics for OH (n = 618, 354 female and 264 male)

Age	n	Median age	Sex		Handedness		
			Female	Male	Left (F/M)	Mixed (F/M)	Right (F/M)
18–29	203	23	120	83	16/10	0/0	104/73
30–39	65	34	32	33	1/2	0/0	31/31
40–49	72	44.5	52	20	4/3	0/0	48/17
50–59	79	56	50	29	3/4	1/0	46/25
60–69	101	64	51	50	6/8	0/0	45/42
70–79	71	74	37	34	2/3	0/0	35/31
80–93	27	84	12	15	0/1	1/0	11/14

**Table 2:** Participant demographics for OHA (n = 513, 289 female and 224 male)

Age	n	Median age	Sex		Handedness		
			Female	Male	Left (F/M)	Mixed (F/M)	Right (F/M)
18–29	163	23	97	66	15/7	0/0	82/59
30–39	56	34	31	25	1/1	0/0	30/24
40–49	58	45	39	19	4/3	0/0	35/16
50–59	65	56	39	26	1/3	1/0	37/23
60–69	87	64	43	44	6/8	0/0	37/36
70–79	65	74	33	32	2/3	0/0	31/29
80–93	19	84	7	12	0/1	1/0	6/11

Participants were recruited from Kingston, Ontario and Calgary, Alberta with test procedures approved by the Research Ethics Boards of Queen’s University and Providence Care as well as the Conjoint Health Research Ethics Board at the University of Calgary. All participants gave their written and informed consent to have their data collected for research purposes.

Healthy human participants completed a checklist (Additional file 1) affirming that they had no neurological or musculoskeletal impairments, had normal or correct to normal visual acuity, and were able to understand task instructions [31, 32].

### Apparatus

**Fig. 1:**
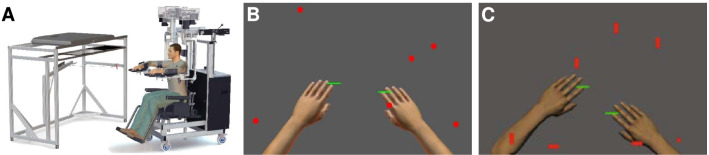
Apparatus and task setup. **A** The Kinarm exoskeleton robot used for both tasks. **B** The virtual workspace for OH. Participants must use the green paddles to hit as many of the 300 red targets as possible. The participant’s arms are not visible during the trial; they are included for illustrative purposes only. **C** The virtual workspace for OHA. Participants must hit as many of the 200 targets as possible while avoiding the 100 distractors. An object’s status as a target or distractor is determined by its shape

For both tasks, participants were seated in a bilateral Kinarm exoskeleton lab (Kinarm, Kingston, Ontario; [36]; Fig. 1A), which provides full gravitational support to the upper limbs while allowing movement in the horizontal plane [31].

### Experimental tasks

The OH task tests an individual’s ability to generate quick and accurate arm movements throughout the workspace. Participants are instructed to use their hands (represented visually as 5 cm green paddles) to hit 300 circular objects (red, 2 cm in diameter) that move towards them in a virtual workspace projected in the horizontal plane (Fig. 1B). Objects are dropped from 10 invisible bins along the horizontal axis, 30 objects per bin, with each bin dropping an object in random order before repeating. Objects are dropped at an increasing rate throughout the trial and with increasing speeds [31]. This results in the task becoming more difficult over time and ensures that all participants end their trial by being overwhelmed.

The OHA task is similar, except that an arbitrary decision rule is introduced based on the red objects’ different shapes (Fig. 1C). Two of the eight shapes are chosen by the task to indicate targets while the remaining shapes are used to indicate distractor objects and these shapes are communicated to the participant at the beginning of the trial. Participants were instructed to hit as many of the 200 targets as possible without hitting the 100 distractor objects [32]. Since OHA is performed in the same virtual workspace as OH and has the same explicit goal, we see it as a more difficult version of OH. This increased difficulty results from the arbitrary decision rule and how it requires neural processes for OHA that are unnecessary in OH, e.g., perceiving shapes and classifying targets vs. distractors. This increased difficulty is therefore a difference in kind and not degree, as with OH’s increased difficulty throughout the trial.

The exoskeleton robot recorded velocities and positions of the hands and objects during both tasks with a sampling frequency of 200 Hz. Contact between paddles and objects was simulated by the exoskeleton robot using a 50 ms force pulse; this feedback was omitted during OHA for paddle/distractor contacts to provide instantaneous feedback to the participant that the distractor was not a one of the two targets. Several parameters were computed once the trial was completed for either task to summarize the participant’s performance (Table 3).

**Table 3:** Parameters recorded by the Kinarm and computed for the OH and OHA tasks [3]

Parameter	Units	Description
Mean hand speed L/R	$\text {cm}/\text {s}$	The participant’s mean hand speed for the left/right hand during the trial.
Mean hand speed bias	$\frac{\text {cm}/\text {s}}{\text {cm}/\text {s}}$	A value from − 1 (all left hand) to 1 (all right hand) which describes the bias in mean hand speed between the hands.
Movement area L/R	$\text {cm}^2$	Area the participant used with the left/right hand during the trial. Determined by a convex hull that encompasses the complete hand path.
Movement area bias	$\text {cm}^2/\text {cm}^2$	A value from − 1 (all left hand) to 1 (all right hand) which describes the bias in movement areas between the hands.
Hand bias of hits	$\text {n}/\text {n}$	A value from − 1 (all left hand) to 1 (all right hand) that quantifies which hand is used more often for hitting targets.
Hand selection overlap	%	The sum of the hand switches for each bin, divided by the number of targets.
Miss bias	cm	Where in the workspace the participant’s misses are biased.
Hand transition	cm	Where in the workspace the participant’s preference for using one hand over the other switches.
Median error	%	Percentage of the way through the task (based on number of targets, not time) where the participant has recorded half of their misses.
Distractor proportion	%	Number of distractors hit as a proportion of the total number of objects (targets + distractors) hit. OHA only.
Object processing rate	Hz	Number of objects (targets + distractors) correctly processed when 80% of the task’s objects have been created. OHA only.
Steady-state rate	Hz	Number of targets hit per second while the participant is overwhelmed.
Targets hit	n	The number of targets hit during the trial.
Task scores	1	A global measure of the participant’s performance, with 0 indicating best performance and increasing values indicating worsening performance. Specifically, the root sum of squares distance of the participant’s Z-scores and Zeta-scores compared to healthy control participants.

Both OH and OHA have been shown to be reliable tests of sensorimotor abilities. Neither task showed any significant learning effect for repeat trials conducted within 15 days of each other, as measured by differences in Z-scores for the measured parameters [21]. The lack of learning effects permitted us to include multiple trials performed by the same participant, giving us 812 OH trials from 618 participants and 683 OHA trials from 513 participants.

### Data analysis

**Fig. 2:**
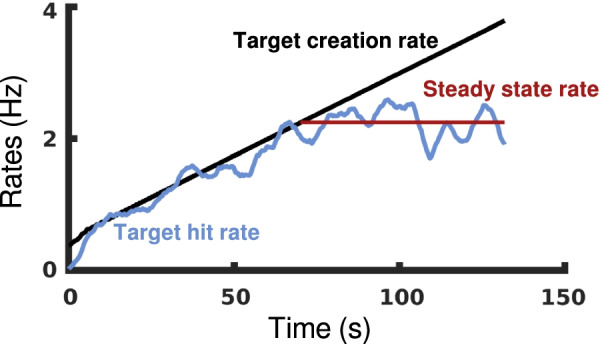
The steady-state rate. Smoothed target creation (black) and target hit (blue) rates are shown for an illustrative participant performing an OH trial. At a certain point in the trial, the participant becomes overwhelmed by the targets being created and reaches a steady-state rate of hitting targets (red)

Values for the parameters in Table 3 were calculated using Dexterit-E Explorer 3.9 (Kinarm, Kingston, ON). The sole exception to this was the steady-state rate, which we observed was a characteristic phenomenon in participants’ trials for both OH and OHA. We automatically calculated the participant’s steady-state rate for both tasks by smoothing the target creation and target hit rates using two iterations of a Kolmogorov-Zurbenko filter with a 5 s sliding window and then determining the last window in which the median difference between the rates was less than 0.1 Hz. A participant’s steady-state rate is their average target hit rate from this window until the end of the trial (Fig. 2). All subsequent analysis was performed using custom scripts in MATLAB (Mathworks Inc., Massachusetts, USA)

We then performed linear regressions between age and each of the parameters in Table 3 for both OH and OHA, recording the slope and intercept parameters for these regressions as well as each parameter’s 95% confidence interval. We also recorded the p-value resulting from Student’s t-test for the null hypothesis that the slope parameter’s value was 0, i.e., that there is no age-related effect.

To assess whether there were any differences in parameter values related to participants’ sex, we divided participants for both tasks according to their self-reported sex. We performed Student’s t-test for each parameter with the null hypothesis that the difference in mean value between males and females was 0. We recorded the p-value for this t-test as well as the 95% confidence interval for the true difference in mean parameter values for male and female participants.

Finally, we tested the age-related declines of parameters with meaningful effect sizes for sex-related effects. We again divided participants for both tasks into two groups according to their self-reported sex and performed separate linear regressions for each group. We then performed a Z-test for each parameter to verify whether the two groups’ regression slopes, *b*, were identical [37]. The Z-statistic was computed according to (1) and we recorded the two-tailed p-value from each test.

1 $$\begin{aligned} Z = \frac{b_{male} - b_{female}}{\sqrt{SE(b_{male})^2 + SE(b_{female})^2}} \end{aligned}$$

To account for the number of comparisons that we performed, we used the Bonferroni correction [38]. Since this study includes 68 comparisons, the significance level 0.05 is corrected to $7.4\times 10^{-4}$.

## Results

### Task validity

There are two aspects of OH and OHA that must be verified in order to confirm that they are suitable for evaluating healthy human participants’ rapid motor behaviours.

**Fig. 3:**
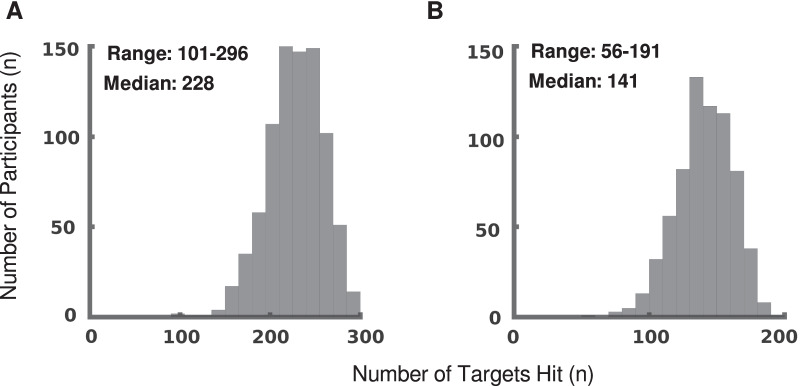
Targets hit. Histograms showing the number of targets hit by participants across all trials for **A** OH and **B** OHA. Note that there are 300 targets in OH but only 200 targets for OHA

First, we want to verify that these tasks are sufficiently challenging that healthy participants must achieve their peak performance in order to do well. The ranges of targets hit in both tasks are within the total number of targets produced and the distributions are roughly normal, with the Kolmogorov-Smirnov test failing to reject the null hypothesis for either task [39]. The width of the distributions, their median values, and the lack of either ceiling or floor effects all indicate that the tasks are capable of capturing a wide range of performance levels in healthy participants (Fig. 3).

**Fig. 4:**
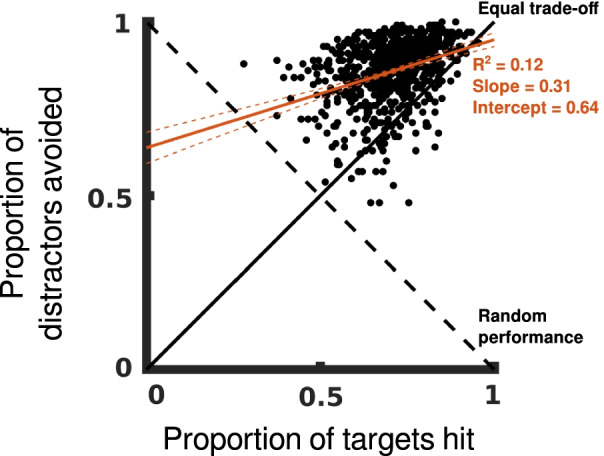
No trade-off between targets and distractors. Scatter plots showing the proportion of targets hit versus the proportion of distractors avoided for OHA. The linear regression line is shown in solid red along with the 95% confidence interval in dashed red. The solid black line indicates equal trade-off between the two objectives, with perfect performance in the top right corner. The dashed black line indicates hitting objects indiscriminately, with no distinction between targets and distractors

Second, we want to verify that participants adhere to task instructions. This is especially necessary for OHA, where two potentially conflicting instructions are given. Scatter-plotting the number of targets hit against the number of distractors avoided shows that most participants were successful in avoiding distractors. There is a positive correlation between hitting targets and avoiding distractors, the linear regression slope is 0.16, telling us that there is no trade-off between the two objectives and that skilled participants were better at both hitting targets and avoiding distractors (Fig. 4). Participants’ relative success at avoiding distractors, seen by the intercept term of 0.64, suggests that this instruction was prioritized during OHA. Taken together, these findings tell us that we can measure success in OHA by the number of targets hit because we do not have to account for variable amounts of effort made by participants to hit targets or avoid distractors.

### No obvious strategies

We analysed participants’ hand trajectories to determine if participants used specific strategies, such as moving both paddles as one unit or alternating between paddles. We quantified this similarity between paddle movements by calculating the cosine similarity where 1 indicates that two vectors are exactly proportional and − 1 indicates two vectors are opposite one another [40]. We calculated the mean cosine similarity between paddle velocities for each second of the trial and then calculated the 50th percentile and the interquartile range of these mean similarities for each trial. The mean cosine similarities between paddle velocities during OH saw a median interquartile range of 0.48 compared with a total possible range of 2. The median 50th percentile of mean cosine similarities was $-0.015$. For OHA, the median interquartile range of paddle velocity cosine similarities was 0.47 and the median 50th percentile was $-0.03$. Visually inspecting the histograms of individual trials’ mean cosine similarities confirmed that these similarities were approximately normal in the distribution. Based on these analyses, we concluded that participants did not use either paddle coordination strategy in OH or OHA.

We also analysed the time between successive object/paddle contacts to determine whether participants alternated between preparation and action. Based on our visual inspection of the gaps between successive paddle contacts, we modelled the gap between two object/paddle contacts as a linear function of the arithmetic mean of the preceding 10 inter-contact gaps, with the scaling factor of 0.965 accounting for the fact that objects are created at an ever-faster rate throughout the trial (Eq. 2).

2 $$\begin{aligned} Gap_i = 0.965 \times \frac{1}{10}\sum _{j = i-10}^{i-1} Gap_j \end{aligned}$$

We then calculated the prediction error for each inter-contact gap as the difference between the predicted and actual values and expressed this as a percentage of that trial’s average inter-contact gap. This simple model’s median prediction error was $0.06\%$ for OH (interquartile range $-0.27\% - 0.47\%$) and $-0.16\%$ for OHA (interquartile range $- 1.37\% - 1.26\%$). Since inter-contact gaps were predictable in both tasks as a simple average of previous gaps, we concluded that participants were continuously trying to maximize their instantaneous rate of object/paddle contacts and did not sacrifice this for longer-term considerations.

### Steady-state rates in rapid motor behaviour tasks

We observed that participants became overwhelmed by the number of targets in the environment for both tasks (Fig. 2). When this occurred, participants hit targets at a steady-state rate regardless of how many additional targets the task produced. This confirms our belief that participants are required to produce peak performance in order to achieve their best results.

**Fig. 5:**
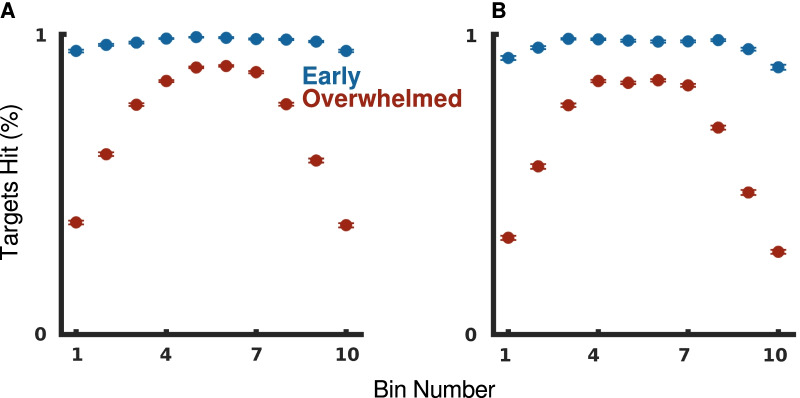
Participants have different per-bin accuracies during different phases of the trial. Mean percentage of targets hit by horizontal bin across all trials for **A** OH and **B** OHA. The standard error of the mean is shown as bars for each point, but largely overlaps with the mean itself. Targets are split according to whether they were created during the participant’s individual early (blue) or overwhelmed (red) phase

We defined the overwhelmed phase as beginning in the last 5 s window where the median difference between target creation and target hit rates was less than 0.1 Hz; the early phase of the task occurs before this when the participant keeps up with the target creation rate. We observed that participants adopted two distinct strategies for these two phases, illustrated by visualizing the mean accuracy of participants according to targets’ horizontal bin location (Fig. 5). In the early phase of the task, participants were able to hit more than 95% of targets, regardless of the targets’ horizontal bin. The limit on the target hit rate during this phase was the task’s target creation rate.

In the overwhelmed phase of the task, a participant’s target hit rate was limited by their own abilities. Trade-offs between speed, accuracy, and effort were required to maximize and maintain the steady-state rate. Not only did participants hit fewer targets during the overwhelmed phase, but they also shifted to a strategy of hitting targets in the central bins while allowing more than 50% of targets in the outermost bins, which required the most time and effort, to pass through the workspace unhit.

**Fig. 6:**
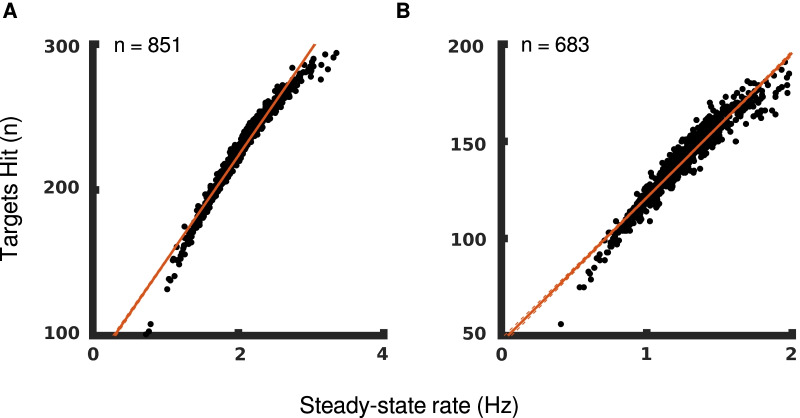
Hits and stead-state rates correlate. Participants’ number of targets hit and steady-state rate are scatter-plotted for **A** OH and **B** OHA. Linear regression lines are shown in red

The instruction to participants across both tasks is to hit as many targets as possible, which makes it the natural way to evaluate how well a participant performed. Figure 6 shows that steady-state rates and number of targets hit correlate very well for both OH and OHA.

### Age-effects in OH and OHA

Tables 4 and 5 report the estimated slope parameters for linear regressions between age and each of the parameters of interest for the two tasks.

**Table 4:** Estimated values and 95% confidence intervals of the slope parameter for linear regressions between age and the parameters listed in Table 3 for OH. Also included are p-values for the null hypothesis that a regression’s slope parameter is equal to 0

Parameter	Slope	95% CI	p
Mean hand speed left	0.0005	(0.0003, 0.0007)	$2.289\times 10^{-6}$
Mean hand speed right	0.0005	(0.0003, 0.0007)	$4.853\times 10^{-6}$
Mean hand speed bias	$-1.172\times 10^{-5}$	(− 0.0002, 0.0002)	0.9215
Movement area left	$1.173\times 10^{-6}$	($-9.401\times 10^{-5}$, $9.636\times 10^{-5}$)	0.9807
Movement area right	$3.731\times 10^{-5}$	($-6.349\times 10^{-5}$, 0.0001)	0.4677
Movement area bias	0.0002	($-9.620\times 10^{-5}$, 0.0005)	0.1994
Hand bias of hits	0.0003	($7.599\times 10^{-5}$, 0.0006)	0.0106
Hand selection overlap	$-8.004\times 10^{-5}$	(− 0.0002, $5.239\times 10^{-5}$)	0.2358
Hand transition	$-8.666\times 10^{-5}$	(− 0.0002, $4.433\times 10^{-6}$)	0.0622
Miss bias	0.0002	($5.478\times 10^{-6}$, 0.0003)	0.0427
Median error	− 0.1058	(− 0.1228, − 0.0888)	$1.279\times 10^{-31}$
Targets hit	− 0.7333	(− 0.8212, − 0.6455)	$2.550\times 10^{-52}$
Steady-state rate	− 0.0099	(− 0.0111, − 0.0087)	$4.150\times 10^{-52}$
Task score	− 0.0020	(− 0.0041, $1.467\times 10^{-4}$)	0.0680

**Table 5:** Estimated values and 95% confidence intervals of the slope parameter for linear regressions between age and the parameters listed in Table 3 for OHA. Also included are p-values for the null hypothesis that a regression’s slope parameter is equal to 0

Parameter	Slope	95% CI	p
Mean hand speed left	− 0.0002	(− 0.0004, $-2.800\times 10^{-5}$)	0.0222
Mean hand speed right	− 0.0003	(− 0.0005, − 0.0001)	0.0015
Mean hand speed bias	− 0.0002	(− 0.0005, 0.0001)	0.2358
Movement area left	− 0.0002	(− 0.0003, − 0.0001)	$3.563\times 10^{-5}$
Movement area right	− 0.0002	(− 0.0003, $-8.192\times 10^{-5}$)	0.0003
Movement area bias	0.0001	(− 0.0002, 0.0005)	0.4462
Hand bias of hits	− 0.0001	(− 0.0005, 0.0002)	0.3678
Hand selection overlap	− 0.0003	(− 0.0004, − 0.0001)	0.0003
Hand transition	$-6.746\times 10^{-5}$	(− 0.0002, $5.309\times 10^{-5}$)	0.2723
Miss bias	$9.184\times 10^{-5}$	($-8.422\times 10^{-5}$, 0.0003)	0.3061
Median error	− 0.1360	(− 0.1558, − 0.1163)	$4.509\times 10^{-37}$
Distractor proportion	0.1307	(0.1112, 0.1502)	$1.960\times 10^{-35}$
Object processing rate	− 0.0100	(− 0.0111, − 0.0089)	$5.772\times 10^{-62}$
Targets hit	− 0.6120	(− 0.6739, − 0.5501)	$3.762 \times 10^{-67}$
Steady-state rate	− 0.0077	(− 0.0085, − 0.0069)	$1.226 \times 10^{-62}$
Task score	− 0.0014	(− 0.0038, 0.0010)	0.2500

We observe that parameters associated primarily with the motor system, such as mean hand speeds or movement areas, show no decrease with age for either task. The only p-values that are significant at the corrected level indicate an increase in mean hand speed for both the left and right hands in OH and a decrease in movement area with the left hand in OHA. These effect sizes are very small: every decade of age results in the mean hand speed for both hands increasing by $0.005 \,\text {cm}/\text {s}$.

$$\begin{aligned} 0.0005 \frac{\text {cm}/\text {s}}{\text {year}} \times 10\,\text { year} = 0.005 \,\text {cm}/\text {s} \end{aligned}$$

Over a 60-year span of adulthood, this would result in a total increase of only $0.03 \,\text {cm}/\text {s}$.

$$\begin{aligned} 0.0005 \frac{\text {cm}/\text {s}}{\text {year}} \times 60\,\text { year} = 0.03 \,\text {cm}/\text {s} \end{aligned}$$

Parameters that decline with age are not clearly associated with the motor system and these parameters have meaningful effect sizes over the course of an average human lifespan. In OH, every decade of life moves participants’ median error forward by an average of 3 objects in the task.

$$\begin{aligned} -0.1058 \%/\text {year} \times 300 \text { obj} \times 10\text { year} = 3.17 \text { obj} \end{aligned}$$

Every decade of life also reduces on average a participant’s OH steady-state rate by 0.1 Hz—6 targets per minute—and reduces their number of targets hit by 7. Over the course of 60 years of adulthood, this results in steady-state rates that are 0.6 Hz lower and 42 fewer targets hit over a single trial, which represents 14% of all available targets.

Participant performance in OHA saw similar declines in these parameters, with every decade of life reducing steady-state rates by 0.077 Hz on average and the number of targets hit by 6. Participants also became increasing worse at avoiding distractor objects, with distractor proportions increasing on average by 7.8 percentage points from age 20 to age 80.

$$\begin{aligned} 0.1307 \%/\text {year} \times 60\text { year} = 7.84 \% \end{aligned}$$

**Fig. 7:**
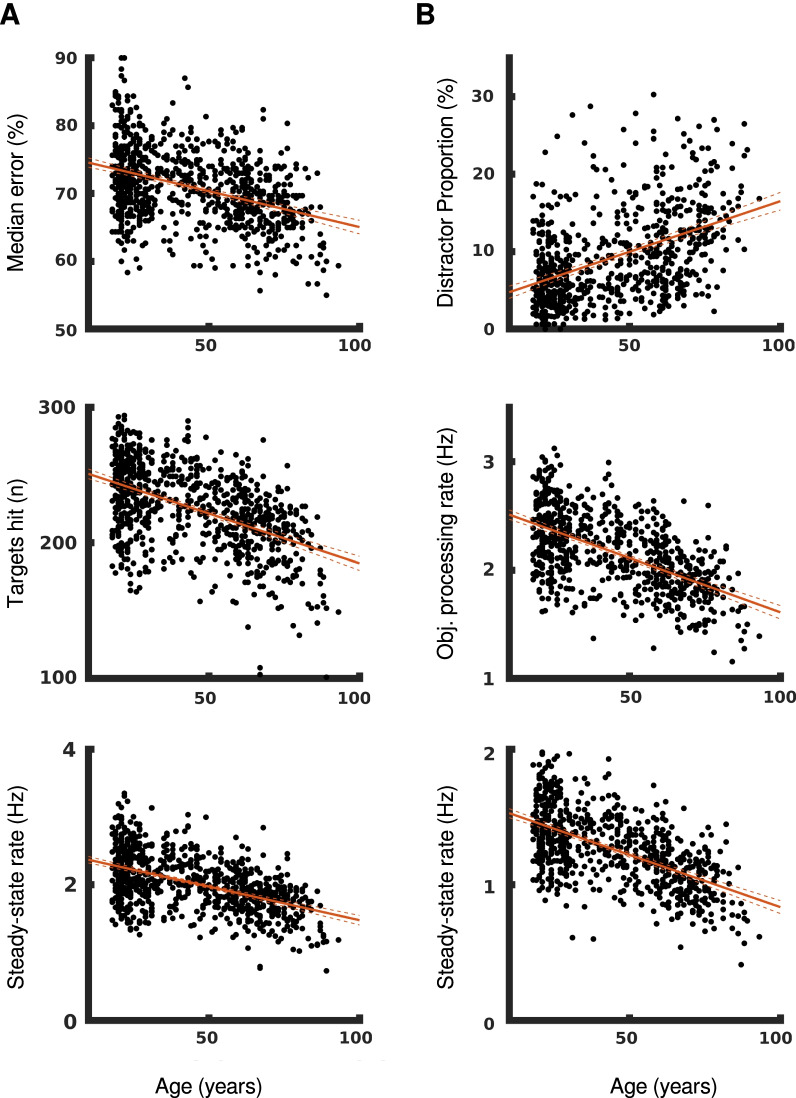
Age-related declines in OH and OHA. Scatter plots between participant age and parameters showing significant aging effects for **A** OH and **B** OHA. Linear regressions are shown in solid red and the regression’s 95% confidence intervals are shown in dashed red

Scatter plots for selected parameters with significant aging effects are shown in Fig. 7.

### Sex-effects in OH and OHA

Table 6 reports the estimated value and 95% confidence intervals for the difference in mean parameter values between male and female participants.

**Table 6:** Population differences and statistical test results for sex effects in both OH and OHA

Parameter	OH		OHA	
	p	95% CI	p	95% CI
Mean hand speed left	0.01528	(0.0020, 0.0190)	0.0041	(0.0032, 0.0169)
Mean hand speed right	0.0367	(0.0006, 0.0184)	0.5264	(− 0.0049, 0.0095)
Mean hand speed bias	0.3546	(− 0.01460, 0.0052)	0.0015	(− 0.0343, − 0.0082)
Movement area left	$1.007\times 10^{-11}$	(0.0101, 0.0180)	0.0002	(0.0036, 0.0116)
Movement area right	$1.2181\times 10^{-8}$	(0.0083, 0.0169)	0.2091	(− 0.0015, 0.0069)
Movement area bias	0.1553	(− 0.0205, 0.0033)	0.0007	(− 0.0424, − 0.0114)
Hand bias of hits	0.1305	(− 0.0187, 0.0024)	0.0202	(− 0.0281, − 0.0024)
Hand selection overlap	0.1500	(-0.0015, 0.0097)	0.0189	(0.0013, 0.0147)
Hand transition	0.7218	(− 0.0032, 0.0047)	0.1063	(− 0.0009, 0.0092)
Miss bias	0.8718	(− 0.0064, 0.0075)	0.7090	(− 0.0059, 0.0087)
Median error	$1.0424\times 10^{-6}$	(1.169, 2.717)	0.5847	(− 1.195, 0.6743)
Distractor proportion	–	–	0.6504	(− 0.6866, 1.099)
Object processing rate	–	–	0.0646	(− 0.0031, 0.1043)
Targets hit	$4.144\times 10^{-11}$	(9.9753, 18.2396)	0.0548	(− 0.0655, 6.3569)
Steady-state rate	$1.110\times 10^{-10}$	(0.1326, 0.2461)	0.0516	(− 0.0003, 0.0828)
Task score	0.6760	(− 0.0722, 0.1112)	0.5732	(− 0.0711, 0.1284)

Two parameters related to the motor system, both of the movement area parameters, show a sex-based effect in OH but the effect sizes are quite small: male participants used an additional $0.1 \text { cm}^2$ with both hands compared to an overall workspace size of $5200 \text { cm}^2$. On the whole, then, there are no practical differences between motor parameters for male and female participants.

Three other parameters show a sex-based effect in OH: median error, number of targets hit, and steady-state rate. All three parameters indicate that male participants hit between 10 and 18 more targets than female participants during an OH trial. This difference is 3–6% of all available targets and 5–9% of the range of targets hit in OH (Fig. 3A). These differences disappear in OHA, however, and we see no sex-based effects on performance in the OHA task for any parameters.

Age-related declines for parameters with significant effects and meaningful effect sizes were tested for any sex-based effects by comparing regression slopes between male and female participants. Aging-related declines were similar for male and female participants (Table 7).

**Table 7:** Estimates of the linear regression slopes for given parameters against age for both male and female participants. The 95% confidence interval for these estimates is reported in brackets. Z-test p-values are reported for the null hypothesis that male and female participants had the same regression slopes for age-parameter regressions

OH			
Parameter	$b_{male}$	$b_{female}$	p
Median error	− 0.1108 (− 0.1371, − 0.0844)	− 0.1118 (− 0.1334, − 0.0903)	0.9503
Targets hit	− 0.7923 (− 0.9151, − 0.6695)	− 0.7584 (− 0.8725, − 0.6443)	0.6908
Steady-state rate	− 0.0111 (− 0.0129, − 0.0094)	− 0.0099 (− 0.0114, − 0.0084)	0.2958

OHA			
Parameter	$b_{male}$	$b_{female}$	p
Median error	− 0.1344 (− 0.1670, − 0.1018)	− 0.1396 (− 0.1645, − 0.1146)	0.8046
Distractor proportion	0.1279 (0.0990, 0.1567)	0.1354 (0.1086, 0.1622)	0.7078
Object processing rate	− 0.0105 (− 0.0121, − 0.0090)	− 0.0101 (− 0.0115, − 0.0087)	0.6902
Targets hit	− 0.6686 (− 0.7630, − 0.5742)	− 0.6014 (− 0.6823, − 0.5205)	0.2879
Steady-state rate	− 0.0084 (− 0.0097, − 0.0072)	− 0.0076 (− 0.0087, − 0.0065)	0.3121

## Discussion

It is well understood that the human sensorimotor system diminishes with age [6–17]. These diminishing abilities are the result of impairments in both the muscular and nervous system, and there is evidence that impairments in the brain and nervous system can lead directly to muscular impairments such as muscle weakness [41]. We systematically studied the effects of age on rapid motor skills with two interactive tasks: OH and OHA. Our results show that an individual’s age-related decline in performance for both tasks is best seen in parameters that we associate with ongoing processes, not the motor system, and that there is no sex-related effect for this decline. This suggests that age-related motor deficits may result from diminished cognitive abilities, such as planning or decision-making, as much as any reduction in strength or coordination. Three parameters showed significant sex effects in OH, but none showed a significant sex effect in OHA.

Both OH and OHA push participants to perform at the peak of their abilities. Although younger participants outperformed older participants in both tasks, this did not result from higher mean hand speeds, larger movement areas, or less bias in hand selection. In fact, the only parameters directly associated with the motor system that showed any significant change with age was the mean speed of each hand and this increased with age. Instead, younger participants derived their advantage in performance from higher steady-state rates: when it came to peak performance, younger participants could sustain a higher rate of hitting targets. These higher steady-state rates are reflective of an ability to perform the OH and OHA tasks at more challenging levels and they occurred without a corresponding increase in mean hand speeds.

Further analysis showed that both maximum and median hand speeds increased slightly with age, but maximum and mean hand acceleration showed no age-effect. We did not consider the increases in hand speeds to be significant due to the small effect size, noting that these higher hand speeds reflect the fact that older participants reach the overwhelmed phase earlier in the task and therefore spend longer making vigorous movement. This extended period of vigorous movement may have contributed to older participants’ poorer performance through a speed-accuracy tradeoff, though there is evidence that older adults will generally sacrifice speed for accuracy in speeded response tasks [42, 43]. Alternatively, this may be evidence that cognitive abilities enable slower, deliberate movements and that older adults are only able to compensate for cognitive declines up to a certain point. This interpretation is in line with the compensation-related utilization of neural circuits hypothesis, which posits that the aging brain’s inefficient processing requires it to recruit more neural resources than a younger brain in order to achieve the same output [44, 45]. It would also be consistent with the scaffolding theory of aging and cognition, which integrates the activation of neural resources in response to aging and challenge into one mechanism [46].

Of course, these are general, population-level results. Some older participants did much better than their age cohort, hitting as many targets as and maintaining steady-state rates similar to participants who were decades younger. It is unclear whether these individuals began at superlative levels of performance before declining with age, or if their functional age has decreased more slowly than their chronological age [47]. This phenomenon is especially seen with “super-agers” who maintain their fitness well into their old age [48]. These older adults’ neuroanatomy shares similarities with the neuroanatomy of younger adults, which supports superior cognitive skills, such as attention and memory encoding, and higher processing speeds compared with other older adults [49, 50]. It is possible that these older participants in the present study have preserved cognitive abilities which allow them to minimize their performance drop over the decades, although a more detailed analysis of this issue is beyond the scope of the present study.

We noted sex-based effects on three parameters during OH, including on the number of targets hit, but not during OHA. This is not due to any additional speed or range of movement by male participants, however, since we found no practical differences in parameters related to the motor system for either task (Table 6). Since the main distinction between the two tasks is OHA’s introduction of distractor objects, we investigated whether participants responded differently based on their sex, but found no evidence to support this idea. A possible explanation is that male participants have trained their sensorimotor skills through their disproportionate representation in both sports, [51–53], and esports [54], with specific evidence existing that female players of action video games have faster visually guided responses than female non-players [55]. These sociocultural explanations have been previously put forward to explain why differences in visuospatial ability general favour male participants [24], though works in this field highlight multiple potential causes across biology and culture [22–24]. The arbitrary decision rule introduced for OHA may have then been sufficiently unfamiliar as to negate this training, an effect that has been reported in high-performance athletes [56]. This is a speculative explanation concerning a population-level result, however, and likely does not capture individual variance. Further study is required to establish the validity of this explanation.

We believe that OH and OHA are both useful for studying interactive motor behaviour. In both tasks, participants must decide about future actions while executing current actions, all while potential targets are continuously created independent of participant action. This “decide-while-acting” paradigm, [34], aligns well with the everyday behaviours that we are most concerned about with aging such as driving or walking along crowded streets.

The freedom to act in an interactive environment also opens the door to ecological interpretations of our results [57]. Participants performing OH and OHA make embodied choices, where action performance is an integral part of decision-making [58, 59]. In embodied cognition, sensorimotor decision problems consist of perceiving affordances in the environment [60] and then selecting from among potential actions through competition [61, 62]. Embodied cognition implies that the sensorimotor system uses an internal model of itself, allowing it to represent and reason about its own abilities and limitations [63]. This is a theoretically sound application of the Internal Model Principle, which states that successful control of a system vulnerable to disturbances requires the controller to incorporate a model of the disturbance and of the system itself [64, 65]. Practically, there is evidence that older adults suffer from a degraded internal model—they are less embodied—and that they compensate for this degraded model by relying on visual feedback in sensorimotor tasks [12]. Thus, the age-related process deficits we observe in this study may be the result of older adults having to respond to unexpected disturbances while being more reliant on external, noisy signals than younger adults who are able to rely on their internal models.Conversely, these deficits may relate to deteriorations in the explicit component of sensorimotor adaptation, which includes strategy formulation and long-term memory [66, 67]. Determining the source of this deterioration is beyond the scope of this study.

A clear finding in our study is the importance of parameters that are not directly linked to the motor system in assessing rapid motor behaviours, which is in line with many views in the sensorimotor literature including: that interactive behavior is produced by the simultaneous processes of specifying and selecting motor actions [57]; that skilled motor performance requires multiple interacting processes to be learned [68]; and that motor actions are the result of hierarchical goal, state, and action processes [69]. Specifically, we highlight the steady-state rate parameter that we uncovered as an indicator of peak performance in both OH and OHA. Reaching a steady-state rate of hitting targets is associated in both tasks with a clear strategic shift to hitting targets in the central part of the workspace, which may be the result of failing to perceive the most lateral targets or deciding to minimize movement costs. Although this question must be left to future study, we believe that measures like the steady-state rate serve as strong support for ongoing, interactive tasks. Unlike reaction time tasks where participants decide and then act, tasks set in interactive environments allow steady-state rates to be used as an ongoing reaction time when measuring participants performance of continuing, interactive behaviours.

As a limitation, our study used only two tasks to assess individuals’ rapid motor behaviours and because OHA was conceived of as an extension to OH, these tasks were related. Our results are therefore specific to this kind of target-hitting task and may not generalize across other motor behaviours. Future studies should seek to replicate these findings using other interactive tasks in order to ascertain whether neural processes are universally implicated in age-related motor decline or whether there are distinct classes of motor behaviours. Additionally, since formal cognitive tests were not administered to screen participants as healthy controls, we cannot ask how cognitive test scores impact the task parameters we recorded. We note, however, that the original papers for the OH and OHA tasks showed relationships between task parameters and certain cognitive test scores, such as the Montreal Cognitive Assessment, Functional Independence Measure, and Behavioural Inattention Test, for participants with stroke [31, 32]. Finally, our study was cross-sectional in nature and not longitudinal. As we covered in our discussion concerning “super-agers,” it is possible that some of our findings reflect idiosyncratic characteristics of our age groups, though the study’s large cohort size should allay these concerns. Regardless, there is evidence that individuals age differently and longitudinal studies may reveal details about age-related declines in rapid motor behaviours that have escaped our notice.

## Conclusions

This study has established that age-related declines in motor abilities can be studied using the OH and OHA tasks specifically and rapid, interactive behaviour tasks more broadly. We found age-related declines in these behaviours consistent with the first part of our hypothesis and our results suggest that these declines have more to do with the brain’s processing abilities than with more basic motor abilities. This is in line with evidence that normal aging is accompanied by reduced processing abilities and may be connected to older adults’ less reliable internal models. In contrast, we found limited evidence to support the second part of our hypothesis that sex affected motor behaviours in the two tasks. We also found no evidence that sex affected the age-related declines that we reported in motor abilities. This work will help the establishment of new neuropsychological tests and equipment for assessing individuals’ motor skills throughout the aging process.

## Supplementary Information

**Additional file 1.** Quantitative Assessment of Arm Movements in Stroke Patients Using KINARM. Volunteer checklistcompleted by all participants to verify their eligibility to be included in the pool of healthy control participants.

## Data Availability

The datasets analysed during the current study are available from Stephen H. Scott (steve.scott@queensu.ca) on reasonable request. All custom MATLAB code used in the analysis of this data is available on Github at 10.5281/zenodo.6110739.

## Abbreviations

OH: Object-Hit
OHA: Object-Hit-and-Avoid
