# Supplementary material for: Quantitatively assessing aging effects in rapid motor behaviours: a cross-sectional study
Source: J Neuroeng Rehabil. 2022 Jul 26;19:82. doi: 10.1186/s12984-022-01035-1 (PMC9327262; doi:10.1186/s12984-022-01035-1)
Supplement: Supplementary file 1 — Additional file 1. Quantitative Assessment of Arm Movements in Stroke Patients Using KINARM. Volunteer checklistcompleted by all participants to verify their eligibility to be included in the pool of healthy control participants. [file 12984_2022_1035_MOESM1_ESM.pdf]

Date Completed: ________________ **Completed By: _______________**

Best Times for appointments:____

Volunteer Contact Information:

Name: __________________________________

Phone: ________________________ **or Email: ___________________________**

Volunteer Script

Thank you for your interest in our study. We are currently conducting a study funded by the Canada Institute of Health Research through Queen' s University and St. Mary's of the Lake Hospital. We are evaluating the use of two types of robotic technology to assess the effects of Stroke on arm function. We are looking for volunteers of all ages, 18 and older, to act as our control group. Participation will require about 2 hours of your time. If you are still interested I would like to ask you a few questions to determine if you are eligible to take part in our study.

Demographic

What is your age? _________

Male / Female (Circle One or Highlight)

Neurological

Y N

☐ ☐ Have you ever had a stroke, mini-stroke or Transient Ischemic Attack (TIA)?

☐ ☐ Have you ever been diagnosed with a disease or condition affecting the brain or spinal cord (eg. Multiple Sclerosis, Parkinson's disease, Huntington's disease, Brain Aneurysm, Brain Tumor, Epilepsy)?

☐ ☐ Have you ever had a brain injury or closed head injury?

☐ ☐ Have you ever suffered a spinal cord injury?

☐ ☐ Have you ever had brain surgery?

☐ ☐ Have you ever had a radiculopathy or nerve root problem in the neck (cervical spine)?

☐ ☐ Have you ever had a brachial plexus injury (injury to a nerve in the shoulder(s)/arm(s))?

☐ ☐ Have you ever had damage to a nerve in the arms (peripheral nerve injury)

(Carpal Tunnel Syndrome would be allowable)?

☐ ☐ Have you ever been diagnosed with neuropathy/peripheral neuropathy/diabetic neuropathy?

Answering YES to any of the above questions excludes subjects from our study. These people should be thanked for their interest and time. If answer is NO to the above questions then move on to next set below. If they have answered YES to any of the questions, confirm that it is not affecting them in regular activities.

Musculoskeletal

Y N

☐ ☐ Have you ever had a fracture to any of the bones of the shoulder, arm or wrist -including the collarbone (clavicle) or shoulder blade (scapula), the upper arm (humerus), the forearm (radius or ulna), the wrist (carpal bones)?

Side (L/R/Both) and bone(s)______________________________

☐ ☐ Do you have arthritis? (Osteo or Rheumatoid or other - circle)

What joint(s) are affected on what side? ____________________________

☐ ☐ Have you ever dislocated your shoulder? **Date and Side (L/R/Both)** _____________________

☐ ☐ Have you ever had a rotator cuff tear? **Date and Side (L/R/Both)**_________________________

☐ ☐ Have you received therapy (eg. physical therapy, massage, chiropractic) for a shoulder problem in the last 3-4 months? **(Y/N)** If Yes - do you still have problem with the shoulder?

Side(L/ R)__________________________

☐ ☐ Have you ever been diagnosed with a frozen shoulder (adhesive capsulitis)?

Date and Side (L/R/Both)___________________________

☐ ☐ Have you ever received a corticosteroid injection for a shoulder problem?

Date and Side (L/R/Both)_____________________________________

☐ ☐ Have you ever had surgery to your shoulders, arms, forearms, wrists or hands? If Yes, Where was the surgery (eg. wrist) and what was the purpose ofthe surgery (eg. Carpal Tunnel release)?

____

☐ ☐ Do you currently have tennis elbow/golfer's elbow (medial or lateral epicondylitis?

Side (LIR/Both)____

☐ ☐ Do you have fibromyalgia or chronic pain affecting you neck, upper back or arms?

Side (L/R/Both)____
